# Residual manifestations of hypercortisolemia following surgical treatment in a patient with Cushing syndrome

**DOI:** 10.1186/s13633-015-0014-2

**Published:** 2015-08-26

**Authors:** Sara K. Bartz, Lefkothea P. Karaviti, Mary L. Brandt, Monica E. Lopez, Prakash Masand, Sridevi Devaraj, John Hicks, Lauren Anderson, Maya Lodish, Meg Keil, Constantine A. Stratakis

**Affiliations:** Department of Pediatric Endocrinology and Metabolism, Texas Children’s Hospital, Houston, TX USA; Division of Pediatric Surgery, Michael E. DeBakey Department of Surgery, Baylor College of Medicine, Texas Children’s Hospital, Houston, TX USA; Department of Radiology, Texas Children’s Hospital, Houston, TX USA; Medical Director of Clinical Chemistry and POCT, Texas Children’s Hospital and Baylor College of Medicine, Houston, TX USA; Department of Pathology, Texas Children’s Hospital and Baylor College of Medicine, Houston, TX USA; Baylor College of Medicine, Houston, TX USA; The Eunice Kennedy Shriver National Institute of Child Health and Human Development (NICHD), NIH, Houston, TX USA

**Keywords:** Cushing syndrome, Obesity, PPNAD, Primary pigmented nodular adrenocortical disease

## Abstract

**Context:**

Cushing Syndrome is difficult to diagnose, and the comorbidities and persistent late effects of hypercortisolemia after treatment of the primary disease are challenging for the patient and the endocrinologist.

**Objective:**

To report the case of a girl with obesity and hypertension, ultimately diagnosed with Cushing syndrome due to primary pigmented nodular adrenocortical disease. In this case, the complications of hypercortisolism persisted short term despite surgical intervention.

**Patient:**

A 4 year old morbidly obese African-American girl with developmental delay presented with hypertensive emergency in the ER and 18-month history of progressive weight gain. Her previous history included premature adrenarche, hypertension, seizures and a random high cortisol with suppressed ACTH. She was subsequently stabilized, and a diagnostic work-up persistently demonstrated elevated cortisol and suppressed ACTH. An abdominal MRI showed bilateral adrenal multinodular disease, consistent with multinodular hyperplasia of the adrenal glands. Based on these findings the patient underwent a bilateral adrenalectomy, which confirmed primary pigmented nodular adrenocortical disease. The patient had a complicated, protracted post-operative course requiring adjustment of therapy for persistent hypertension. Two months after surgery, she was readmitted to the Emergency Department with hyperpyrexia and hypertension and succumbed to the complications of sepsis.

**Conclusions and outcome:**

This case highlights the significant diagnostic and therapeutic challenges in treating children with Cushing syndrome. Resolution of the source of hypercortisolemia does not imply regression of hypertension or recovery of the immune system. Although the child underwent bilateral adrenalectomy, persistent consequences of prolonged severe hypercortisolism contributed to her death two months later.

## Background

Cushing syndrome, a syndrome of excessive cortisol circulation, is a rare diagnosis in the pediatric population and may arise from either endogenous secretion or exogenous and iatrogenic administration of glucocorticoids [1]. Significant overlap of symptoms, such as weight gain, hypertension, and glucose intolerance may occur, rendering the diagnosis difficult to establish, especially in the case of mild symptoms [2–4]. The most common cause of Cushing syndrome is administration of glucocorticoids, which are being used more commonly in a wide range of childhood diseases [5, 6]. Endogenous Cushing syndrome is very rare, particularly in pediatric patients [4]. The two major classifications of Cushing syndrome in pediatrics are those with ACTH-independent and ACTH-dependent causes, and the various etiologies appear to be somewhat age-dependent [1, 7]. Of the ACTH-independent etiologies, which account for 15 % of pediatric cases, the major ones include exogenous steroid use, adrenocortical tumors, and primary adrenocortical hyperplasia [1, 5]. Regardless of the underlying cause, Cushing syndrome is arguably one of the most complex endocrine conditions to diagnose [8]. The disease creates short and long-term, significant consequences even if the source of the hypercortisolemia is eradicated. Cushing syndrome is also difficult to treat, and a full cure with complete return to normalicy occurs infrequently. Herein, we present a unique case of a very young child with primary pigmented nodular adrenocortical disease (PPNAD), resulting in Cushing syndrome and several co-morbidities that persisted after treatment of the primary condition.

## Case

### Presentation

A 4 year, 4-month-old, morbidly obese, African-American female with developmental delay and microcephaly presented to our Emergency Department (ED) with hypertensive emergency.

### Diagnosis evaluation

**Fig. 1:**
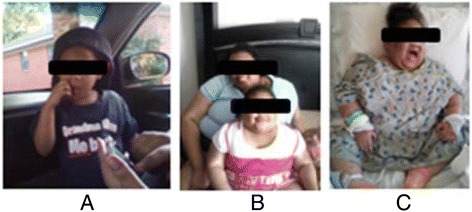
Photographic progression demonstrating the dramatic weight gain of our patient over 18 months. **a**) 18 months prior to presentation, **b**) Just prior to presentation with her mother and **c**) At admission while in the ICU

**Fig. 2:**
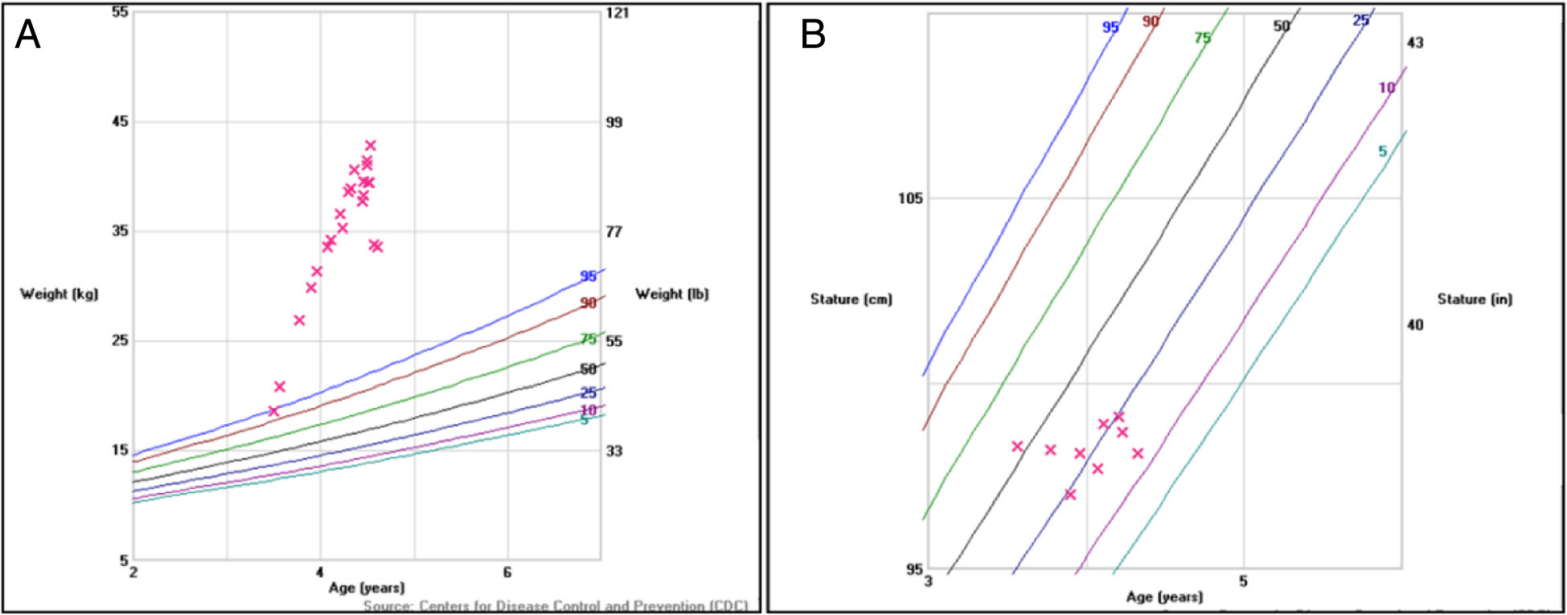
Height and Weight curve over 18 months for our patient, showing a dramatic increase in weight from the 95^th^ percentile to well beyond, yet a negligible height velocity

The inpatient endocrinology service was consulted for a diagnostic work-up for Cushing Syndrome given a previous elevated cortisol value and cushingoid features. Her rapid weight gain was documented in photographs provided by the family (Fig. 1). Physical examination revealed poor growth (height of 98.2 cm, unchanged in 7 months, Fig. 2), central obesity (weight, 40.6 kg; BMI >99^th^ percentile, Fig. 2), moon facies, dorsocervical fat pad, and prominent skin folds on all extremities. There was no palpable glandular breast tissue. Striking violaceous striae were present on the thighs and axilla. She did not have any areas of increased skin pigmentation. Normal female genitalia for age were identified. Sparse Tanner II pubic hair extended from the labia to the pubis.

Further testing revealed a midnight salivary cortisol concentration of 0.341 ug/dl (normal, <0.010-0.090 ug/dl) and two 24-hour free urinary cortisol concentrations of 67 ug/d and 213.3 ug/d (normal, <18 ug/d). Serum cortisol and ACTH concentrations were measured throughout the day. Her serum cortisol was consistently elevated (range, 12–28.2 ug/dl) with no diurnal variation, and her ACTH was consistently suppressed (<5 pg/ml). Her serum 11-deoxycortisol concentration was elevated at 661 ng/dl (normal, 7–210 ng/dl), but she demonstrated normal concentrations of 17-hydroxyprogesterone, deoxycorticosterone, dehydroepiandrosterone, and dehydroepiandrosterone sulfate. Serum androgens were borderline elevated, with androstenedione of 65 ng/dl (normal 5–51 ng/dl) and testosterone of 10 ng/dl (normal <9 ng/dl).

**Fig. 3:**
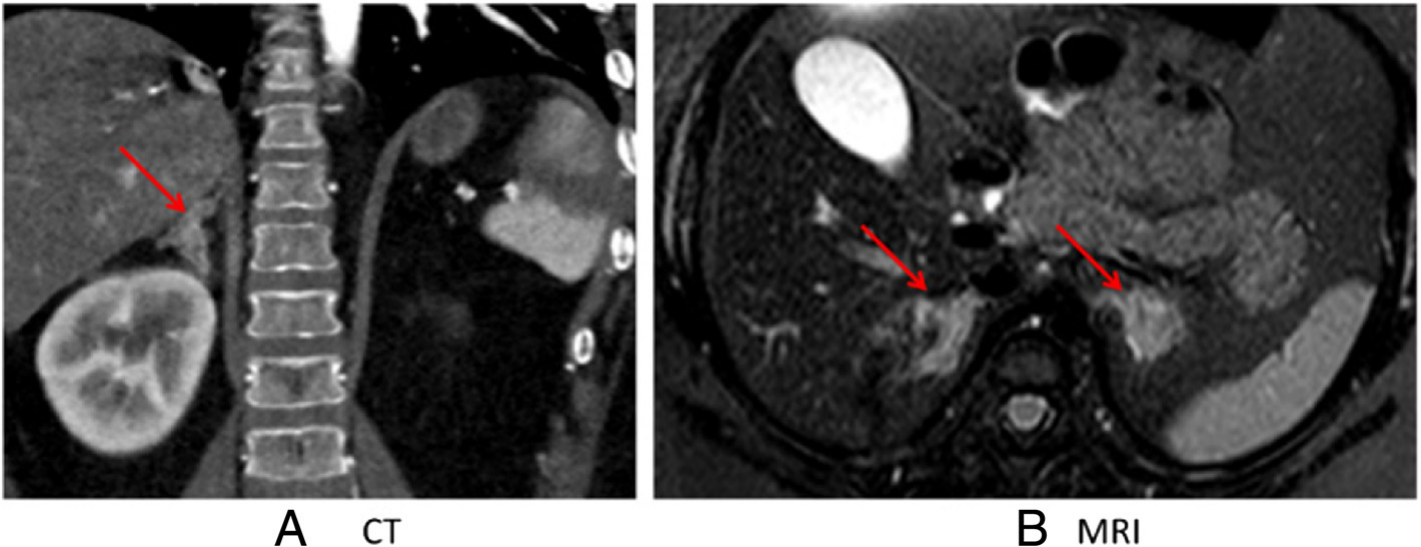
**a** CT angiogram of the abdomen demonstrating thickened limbs of the adrenal glands with nodular contour. Arrows point to adrenal glands. **b** Magnetic resonance imaging (MRI) of the abdomen confirmed the presence of thickened nodular adrenal glands with no evidence of a dominant nodule to suggest adenoma or adrenal cortical carcinoma. Arrows point to the two adrenal glands

Imaging studies obtained included an MRI of the brain showing no increase in the adenohypophyseal size, and no evidence of a pituitary adenoma. CT scan of the abdomen revealed diffusely thickened limbs of both adrenal glands, with a nodular contour. MRI was performed in an attempt to get a more discriminating view of the adrenal glands. (Fig. 3). MRI again demonstrated uniformly thickened adrenal glands with no evidence of an adenoma or adreno-cortical carcinoma. A skeletal survey was negative for fibrous dysplasia. Bilateral breast ultrasound and echocardiogram were negative for myxomas and fibromas.

### Management

Based on the combination of her physical examination, laboratory evaluation, and imaging studies, a multidisciplinary conference was held to discuss further intervention. A diagnostic and therapeutic bilateral adrenalectomy was scheduled 3 weeks into admission following completion of antibiotic therapy for pneumonia, a urinary tract infection, and subsequent culture-negative febrile episodes that occurred during her hospitalization. Her laparoscopic adrenalectomy was converted to an open procedure secondary to poor visualization and challenges of her body habitus. Intraoperatively, she received her initial dose of glucocorticoid replacement therapy (100 mg of hydrocortisone).

**Fig. 4:**
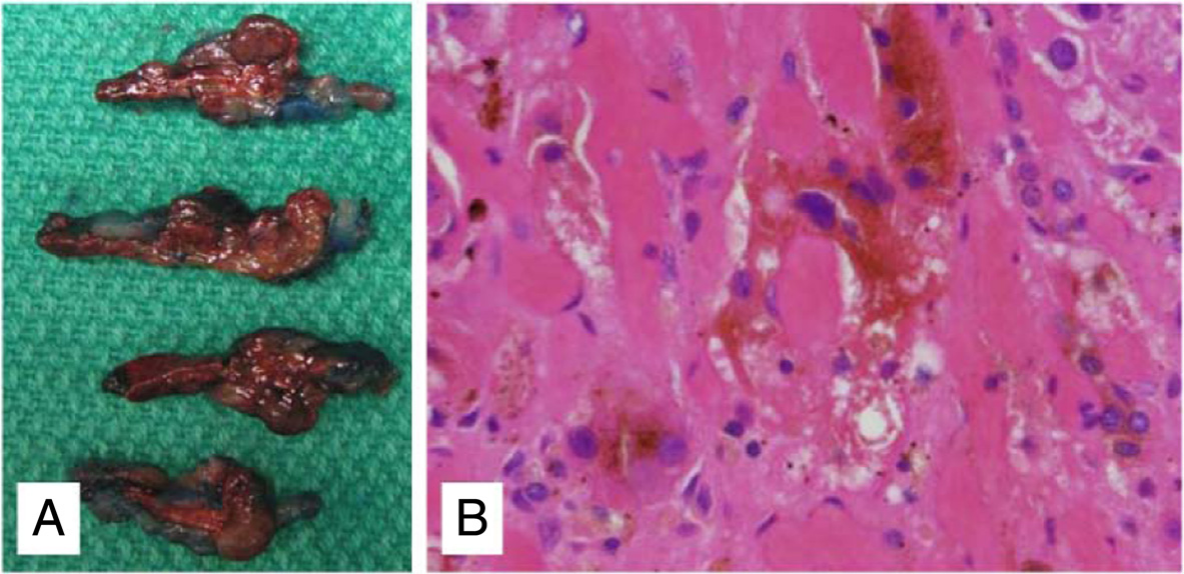
**a** Adrenal gland on cross sectioning shows several discrete, deeply red-brown nodules, consistent with PPNAD. **b** Golden brown pigment with the cytoplasm of cortical cells from the adrenal gland are seen on microscopic examination, imparting the typical pigment seen on gross examination with PPNAD (H&E stain, original magnification 400x)

Upon gross examination, the left adrenal gland weighed 4.1 grams and the right adrenal gland weighed 4 grams (expected weight of combined adrenal glands 4.9 +/− 2 grams). Both adrenal glands demonstrated multiple cortical-based, deeply red-brown pigmented nodules, with intervening atrophic adrenal cortex (Fig. 4a). Microscopic examination of both the right and left adrenal glands showed features consistent with primary pigmented nodular adrenocortical disease (Fig. 4b). The adrenal glands showed nodules with markedly enlarged cortical cells with abundant eosinophilic cytoplasm and golden-brown pigment, characteristic for PPNAD.

Our patient had a complicated, protracted postoperative course. She remained hypertensive, requiring multidrug therapy. She developed persistent fevers, despite receiving broad-spectrum antibiotic coverage. She developed two superficial surgical site infections, one requiring percutaneous drainage and the other incision and drainage with negative pressure wound therapy. In addition, she developed an acute nonocclusive thrombus of the distal inferior vena cava extending into the left common iliac vein, which required long-term anticoagulation. After a prolonged hospital course of 61 days, she was discharged home in stable condition with adrenal replacement therapy (hydrocortisone 10 mg/m^2^/day and fludrocortisone 0.05 mg) and several antihypertensive medications (including amlodipine, lisinopril, clonidine patch, and, as needed, nifedipine).

One month after discharge and two months after surgery, our patient returned to the ED quite edematous with septic shock secondary to a life-threatening urinary tract infection. The electronin medical records are not clear concerning whether she received stress dosing of steroids and a cortisol concentration was not obtained. Blood and urine cultures subsequently returned positive for *Klebsiella pneumoniae* and *Enterococcus faecalis*. She succumbed to sepsis despite receiving aggressive treatment and resuscitation in the ED 3 h after presentation. An autopsy was not performed, at the request of her parents.

## Discussion

Our patient presented with glucocorticoid excess and a variety of features including excessive weight gain and decreased linear growth. Glucocorticoid excess can be associated with decreased growth hormone (GH) secretion [9], tissue resistance to IGF-1 [10], and decreased GH response to stimulation testing [9, 10]. Bone age often is delayed, as well [11]. The growth axis may be suppressed for years after cure, leading to inadequate catch-up growth and compromised final adult height, particularly in the peripubertal child [9]. However, it was the hypertension which brought our patient to seek medical attention which eventually led to the diagnosis of PPNAD.

PPNAD is a rare genetic disorder with autonomously functioning nodules that produce excessive cortisol [5]. The finding of these nodules with surrounding areas of atrophy on microscopic examination is pathognomonic for the disease [12]. The atrophy occurs because the autonomous function of the nodules results in suppression of ACTH secretion [1]. PPNAD often is seen in association with Carney complex, a syndrome of multiple neoplasia of the endocrine gland, skin abnormalities, and tumors. It was first described by J. Aidan Carney in 1985 in a case series of 40 patients [13, 5, 14]. Carney complex is inherited in an autosomal dominant manner, with very high penetrance [12]. The current diagnostic criteria include skin lentigines, myxomas, and other endocrine and nonendocrine tumors [15]. Generally, this disease occurs in late adolescence or early adulthood, but a few case reports describe it in younger children [7, 16]. Inactivating mutations in the regulatory subunit of type 1 alpha of the protein kinase A (PRKAR1A) gene have been identified in most of the patients with PPNAD [1, 13]. Protein kinase A (PKA) is the main mediator of cAMP signaling. PRKAR1A functions as a tumor suppressor [13]. Forms of PPNAD without manifestations or family history of Carney complex are rare. Genetic studies were sent and negative for the PRKAR1A mutation. Our patient underwent whole exome sequencing (WES), but a molecular diagnosis has not yet been achieved.

Cushing syndrome is a progressive disease with multisystem involvement. Treatment reverses, but may not completely normalize, many of the deleterious effects of hypercortisolism short term or long term [4]. Hypertension is a presenting feature in Cushing syndrome and brought our patient to medical attention. The cause of hypertension in Cushing syndrome is multifactorial and includes priming of the vessels with glucocorticoids so rendering catecholamines more effective [17], salt-retention from high cortisol state, and variable renin-angiotensin system activation [18]. Hypertension reshapes the cardiovascular system, which does not appear to return to normal, and a permanent loss of vessel elasticity is reported in adults [18]. Data on the long-term outcomes of hypertension are lacking, but some reports indicate blood pressure may not completely normalize [19, 20]. In a review of 113 children with Cushing syndrome, blood pressure normalized in most by one year after cure, but a portion remained hypertensive [21]. Although we have only two months of post-operative data on our patient, her hypertension persisted post-operatively and the patient required multidrug therapy for this comorbiditiy.

In Cushing syndrome, another potential insult to the cardiovascular system is the increased risk of venous thromboembolism (VTE) [22–24]. This increased hypercoagulability appears to be especially problematic after surgery or during active disease [25]. A large review, primarily of adult patients, demonstrated that there is a significantly enhanced risk of postoperative VTE that seems to be related to glucocorticoid excess resulting in modification of coagulation and fibrinolysis [22]. Unfortunately, at this time, no clear consensus guidelines exist for optimal prophylaxis regimens, especially in pediatrics [23]. Our patient did develop a venous clot, likely the result of the hypercoagulable state recognized with Cushing syndrome.

Our patient’s ultimate presentation was presumably fulminant sepsis that was the result of several factors: possible early clinical signs of illness that were not recognized in her home setting that delayed her presentation to the ED; her prolonged hypercortisolemia; her likely being an immunocompromised host; uncertainity regarding whether she received her stress dose of steroids during her acute illness; and adrenal crisis, which is a very frequent complication in the post-adrenalectomy patient that can lead to premature death [26]. Studies have shown that insufficient patient education can be contributory [27]. The increased risk of infection appears to be present prior to treatment and may persist during long-term follow-up [28]. Synthetic glucocorticoids are the most commonly prescribed class of immunomodulatory medications, and supraphysiologic endogenous glucocorticoids can cause a state of immunosuppression [29, 30]. Glucocorticoids represent the endogenous mechanism to suppress inflammatory gene response. Persistent hypercortisolism results in lymphopenia, both directly and indirectly [30]. The immune system was fragile in our patient during times of hypercortisolemia and continued to be so post-operatively. Whether the immune system recovers, and if so, when, remains unclear. Chemoprophylaxis with antibiotics might be prudent, and guidelines for this management are greatly needed.

## Conclusions

This case highlights the numerous significant challenges, both diagnostic and therapeutic, physicians face when treating children with Cushing syndrome and its comorbidities [31, 32]. In addition, it describes the many gaps that may exist in the provision of care for a patient with this condition. These gaps need to be anticipated and taken as seriously as taking the patient to surgery. Although we are focusing on the operating procedure to resolve the hypercortisolemia, we are far from achieving resolution of the cormobidities, regardless of the success of the adrenalectomy. Our patient underwent bilateral adrenalectomy, however the consequences of hypercortisolism were not immediately reversed, similar to cases reported in the literature, particularly for the short term. The resolution of the source of hypercortisolemia does not imply regression of hypertension or recovery of the immune system [21, 28]. Instead, strategies must be developed to deal with both short- and long-term residual manifestations of Cushing syndrome, such as cardiovascular comorbidities and immunosuppression.

Our patient taught us several lessons. First and foremost is that the earlier the diagnosis, the less exposure the patient has to steroids. Accordingly, as has been noted previously, the constellation of clinical signs (including obesity coupled with growth failure) must be evaluated, rather than focusing exclusively on the obesity. The second important lesson is that recognizing the preparedness of the parents to deal with comorbidities of their child’s Cushing syndrome is of paramount importance. The parents’ failure to recognize signs of sepsis combined with most likely lack of stress steroid coverage may have placed our patient at risk for compromise of her immune system and presumably contributed to the decompensation secondary to sepsis. Development of multidisciplinary team guidelines for postoperative management of Cushing syndrome and recommendations for interventions regarding the immune system, as well as on-going family education, would enhance the physician’s success to complete the circle of management challenges and stabilize the patient with Cushing syndrome for the long term.

Consent for photography and use in a manuscript was discussed and obtained from family.

## Abbreviations

BMI: Body mass index
ACTH: Adrenocorticotropic hormone
CT: Computed tomography
MRI: Magnetic resonance imaging
PPNAD: Primary pigmented nodular adrenocortical disease
ED: Emergency department
SBP: Systolic blood pressure
DBP: Diastolic blood pressure
ECHO: Echocardiogram
AST: Aspartate Aminotransferase
ALT: Alanine Aminotransferase
HVA: Homovanillic acid
VMA: Vanillylmandelic acid
IVC: Inferior vena cava
WBC: White blood cell
UA: Urinalysis
BUN: Blood urea nitrogen
